# Capacity building for healthcare workers on preventing and managing female genital mutilation: Impact on knowledge, attitudes, skills, and quality of care—A systematic review

**DOI:** 10.1002/ijgo.70757

**Published:** 2026-01-26

**Authors:** Chioma Oringanje, Sidney Oparah, Christina C. Pallitto, Anthony Okoro, Mavis Otonkue, Faithman Ovat, Ogonna Nwankwo, Martin Meremikwu

**Affiliations:** ^1^ Department of Biology Xavier University Cincinnati Ohio USA; ^2^ Cochrane Nigeria Calabar Institute of Tropical Diseases Research & Prevention Calabar Nigeria; ^3^ Department of Internal Medicine University of Calabar Calabar Nigeria; ^4^ UNDP/UNFPA/UNICEF/WHO/World Bank Special Programme of Research, Development and Research Training in Human Reproduction (HRP), Department of Sexual and Reproductive Health and Research World Health Organization Geneva Switzerland; ^5^ Department of Community Medicine University of Calabar Calabar Nigeria; ^6^ Department of Pediatrics University of Calabar Calabar Nigeria

**Keywords:** female genital mutilation, female genital mutilation/cutting, healthcare professionals, training

## Abstract

**Background:**

Despite declining prevalence in some regions, female genital mutilation (FGM) remains a major public health issue, causing both immediate and long‐term health complications.

**Objectives:**

The objective of this present study was to determine the effect of training healthcare workers, providing access to resources for capacity‐building, such as job aids, and its impact on knowledge, skills, and attitudes toward FGM and the quality of healthcare service delivery.

**Search Strategy:**

The following major databases were searched from inception to May 2023: CINAHL Plus, IRIS, MEDLINE, PsycINFO, SCOPUS, and Web of Science, without language restrictions.

**Selection Criteria:**

Controlled studies based on predefined objectives.

**Data Collection and Analysis:**

Studies were independently assessed for eligibility and risk of bias. Data were extracted for meta‐analyses and the evidence assessed using the GRADE approach.

**Main Results:**

Eight studies, including one cluster randomized controlled trial (RCT), were included The RCT showed moderate‐quality evidence that training plus information, education, and communication (IEC) materials significantly improved knowledge, care practices, and confidence compared to IEC materials alone (*P* < 0.001). Similar trends were reported in the observational studies (very low‐quality evidence). Point‐of‐care flip chart visual aids helped providers communicate messages more effectively to clients.

**Conclusions:**

This review found that providing FGM training to healthcare workers, in addition to capacity‐building resources, may improve knowledge, care for women and girls with FGM, communication skills, and reduce their support for the practice. However, the limited number of studies and the overall low quality of evidence weaken the strength and limit the generalizability of the findings.

## 1 INTRODUCTION

### 1.1 Background

Female genital mutilation (FGM) involves altering female genitalia for cultural or nonmedical reasons.^1^ Recent evidence shows a decline in FGM prevalence among girls aged 16–19 years in many affected regions, especially in parts of West and East Africa and some countries in the Middle East and South Asia.^2^ However, FGM remains a major public health issue, with over 230 million girls and women in 31 countries having undergone the practice.^2^, ^3^ It is also reported in high‐income countries due to international migration.^4^ FGM is typically performed with sharp objects by individuals such as elderly women and traditional birth attendants.^5^, ^6^ Some healthcare providers also perform FGM, citing harm reduction, better hygienic conditions, social pressure from families, or financial gain.^3^, ^7^, ^8^

FGM is practiced on girls aged 4–12 years but may occur days after birth to just before marriage, depending on local traditions.^6^ It is driven by social pressures, religious beliefs, and the belief that it reduces sexual desire. Despite having no health benefits, FGM poses immediate risks, such as pain and bleeding, and long‐term complications, including gynecological and obstetric issues, urinary problems, psychological trauma, and sexual dysfunction,^3^ underscoring the urgent need to eliminate the practice.^9^, ^10^

FGM poses both short‐ and long‐term challenges, complicating the provision of care services for affected women and adolescents. Healthcare providers often struggle to discuss FGM, and some may lack the clinical skills needed, particularly for managing cases involving deinfibulation.^11^ Studies highlights the challenges healthcare workers face in caring for women with infibulation during childbirth, including the need for deinfibulation and managing related complications.^12^, ^13^ In both high‐ and low‐prevalence countries with affected populations, including the United Kingdom, Spain, and the United States, healthcare workers often have significant gaps in theoretical knowledge and practical skills. Some report fear, limited experience, and discomfort discussing FGM.^14^ To address this, WHO has supported priority countries in developing comprehensive action plans, including capacity building for healthcare workers based on evidence‐based resources.^15^

Various activities implemented by ministries of health, international organizations, educators, and nongovernmental organizations have focused on capacity building of healthcare workers to address FGM‐related complications. These include training on legal frameworks, evidence‐based management of sexual, obstetric, and psychosocial complications, and developing competencies in person‐centered communication (PCC) and respectful care.^16^, ^17^, ^18^ Educational approaches range from health talks, visual aids, role‐plays, case studies, and group discussions to hands‐on demonstrations, storytelling, and mobile apps.^17^, ^18^, ^19^ WHO has also developed tools, such as training materials, guidelines, and a clinical handbook to strengthen healthcare capacity and improve care for at‐risk or affected individuals.^19^, ^20^

These resources aim to enhance healthcare workers' knowledge, skills, and confidence in FGM prevention and care, empowering them to engage in informed PCC and influence clients to abandon the practice. In addition, tools such as structured interview guides, infographics, and counseling checklists can help bolster the retention of information among healthcare workers and serve as practical reminders during service delivery.^21^

This review sought to determine the impact of providing FGM training and education to healthcare workers and/or provision of capacity‐building resources such as job aids, information, education, and communication (IEC) materials, clinical guides/handbooks, and other training materials on FGM to healthcare providers on their knowledge, attitudes to FGM, and their skills in providing care for girls and women living with FGM.

## 2 METHODS

The review was conducted in line with the Cochrane Handbook for Systematic Reviews of Interventions version 5.1.0,^22^ and the findings were reported in accordance with the Preferred Reporting Items for Systematic Reviews and Meta‐Analyses (PRISMA) guidelines.^23^ The protocol was registered with the International Prospective Register of Systematic Reviews (PROSPERO; ID: CRD42023426370).

### 2.1 Literature search

A systematic search was carried out of the following electronic databases from inception to May 2023: CINAHL Plus, IRIS, MEDLINE, PsycINFO, SCOPUS, and Web of Science. The full details of the search are detailed in Table S1. We also reviewed the reference lists of relevant studies, including contacts of experts in women's health for additional references. No language restrictions were applied.

### 2.2 Study selection

Two authors independently screened titles, abstracts, and full texts of relevant articles using the Covidence software.^24^ Discrepancies were resolved through team discussion. We considered randomized, non‐randomized studies, cohort studies, case–control studies, and pre‐ and post‐test studies with/without comparative analysis. Any intervention providing information on FGM practices, risks, and care, regardless of delivery mode among healthcare workers, was considered. Outcomes assessed included improved FGM knowledge, communication skills, complication management, attitude change, community education, reduced stigma, and increased reporting.

### 2.3 Data collection

Three authors independently extracted data using a template developed in Microsoft Excel. Where available, we extracted information on location, race, occupation, gender, religion, education, and socioeconomic status, along with healthcare providers' categories. Details included the nature of intervention including job aids provided, delivery method, duration, follow‐up length, and outcomes. Two review authors independently assessed study bias using the Cochrane Risk of Bias tool for Randomized Controlled Trials (RCTs)^22^ and the Cochrane Risk Of Bias In Non‐randomized Studies of Interventions (ROBINS‐I) for non‐randomized studies.^25^ Judgments of “yes”, “no”, and “unclear” were used to indicate a low, high, or unclear risk of bias. Disagreements were resolved through discussion between review authors.

### 2.4 Data analysis

Statistical analyses were performed with Review Manager Web.^26^ Odds ratios (ORs) were reported with 95% confidence intervals (CIs). When percentages were provided, actual numbers were calculated. Individual outcome findings were illustrated using forest plots. When an analysis was not possible, a narrative summary was presented following Synthesis without Meta‐analysis (SWiM) guidelines.^27^ Due to variations in study types, populations, durations, and outcome reporting, data pooling was not feasible. The quality of the evidence was evaluated using the GRADE approach and summarized in GRADE evidence tables (Table S2).

## 3 RESULTS

**FIGURE 1:**
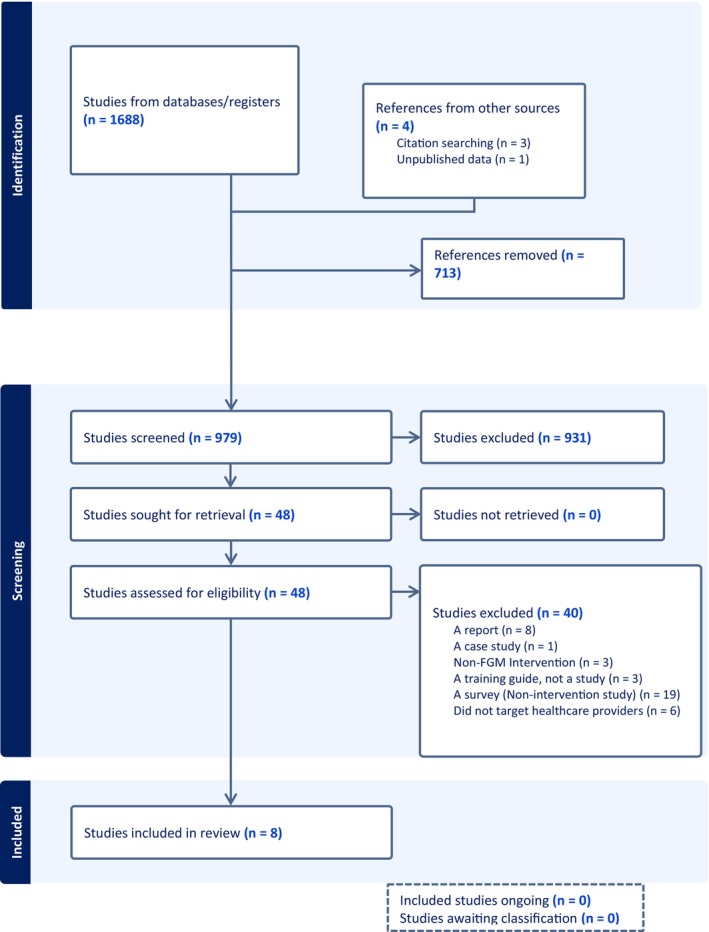
Flow diagram of included studies.

The search returned 1692 articles. After de‐duplication, the titles and abstracts of 979 records were screened, of which 48 were eligible for full‐text assessment and eight studies were included. The included studies are described in Table 1. The excluded studies and the reasons for exclusion are described in Table 2. Details of the search results are presented in a PRISMA flow diagram (Figure 1).

**TABLE 1:** Characteristics of included studies.

Study ID	Method	Participants	Intervention	Outcome reported
Balde et al. (2024)^8^	Cluster RCT	232 ANC providers: 193 (83%) were women, mean years of professional experience 8 ± 7 years; 21 (5%) had a certificate, 158 (68%) had a diploma, 44 (19%) had a Bachelors degree, 1 (0.4%) had a Masters degree and above. 103 (44%) providers were midwives, 51 (22%) were nurses, 54 (23%) were nurse‐midwives, 24 (10%) were others. 37% had received formal training on FGM during clinical training and 54% had undergone FGM. Setting: Antenatal clinics in Guinea, Kenya, Somalia	Experimental group: Intervention was provided at two levels Level 1: Passive distribution of Ministry of Health directives or policy to health facilities on FGM prevention and care, WHO FGM clinical guidelines, a clinical handbook on FGM, and information and educational material in the form of posters to be hung at the health facilities. The materials were distributed without any capacity building to accompany their distribution. Level 2: A PCC training package on FGM prevention. This is an interactive training specifically targeting ANC providers to build their knowledge of FGM, enable them to question their FGM‐related values and attitudes, and build their skills in counseling for FGM prevention using a PCC approach, which is a component of person‐centered care that ensures that the perspectives and preferences of individuals, carers, families, and communities are at the center of decisions and that they have the information and support needed to make decisions. ANC providers were trained to apply a series of structured steps in which they would: “Assess” their client's views on FGM, address and challenge her “Beliefs”, encourage “Change”, and together with the client, “Discuss and Decide” (ABCD). *Control group*: Received Level 1 intervention at baseline	Provide appropriate FGM‐related prevention and care services Correct FGM‐related knowledge responses (ANC providers' mean knowledge score) Appropriate interpersonal communication skills (ANC providers' mean communication skills score) Attitude towards FGM (ANC providers' mean FGM attitude score) Support FGM Support medicalized FGM
Diop et al. (1998)^28^	Pre‐ and post‐intervention study with control arm	Service providers at health centers (nurses‐in‐training or nurse aides, midwives, traditional birth attendants, and health technicians). *N* = 108; experimental arm = 59, controls = 49. Setting: Health centers in Mali (8 = experimental; 6 = control)	Recall of female anatomy and FGM, its context and local rationale, prevalence in Mali and other regions, and the different types of cutting. Emphasis on health‐related FGM complications and their treatment. It also involved supervision of experimental sites by representatives of other organizations directing the research: DSFC, DRSP, and ASDAP and exposing clients at experimental sites to FGM‐related IEC sessions or counseling. The introduction of FGC‐related IEC activities (the use of IEC visual aids such as flip charts, posters, dummies, AV tools) within health talks and consultations at the clinics and during individual consultations with clients. *Control arm*: No specific intervention	Knowledge of FGM types; knowledge of FGC immediate and long‐term complications; providers' attitudes towards FGM; providers' opinion on FGM/C and health (does FGM poses health risk if carried out in a hygienic environment); providers' communication skills and confidence to provide FGM prevention and care to their clients
Newman and Nelson (2003)^29^	Pre‐ and post‐intervention study	RH providers Setting: Hospital in Maine, USA	PRIME II assisted a Ministry of Health (MOH) and local NGO technical working group in developing and field‐testing a national FGC curriculum (the curriculum is part of a FGC resource package for providers, which includes job aids and a 35‐min video that helps providers understand and identify complications from FGC), which was used to train 120 RH providers in Koulikoro and Bougouni districts and Bamako Commune I	Providers' knowledge about prevention and management of FGC complications; providers' attitude towards FGM; providers' confidence in patient counseling
Jacoby and Smith (2013)^17^	Pre‐ and post‐intervention study	All member midwives (CNMs) of the Maine Chapter of the ACNM in active practice (*N* = 11). Setting: USA	An education program was developed that included didactic information, case studies, a cultural roundtable, and a hands‐on skills laboratory of deinfibulation and repair. A laminated procedure card describing the procedure was given to each study participant	Knowledge of FGM; providers' confidence in patient's counseling; confidence to practice deinfibulation and repair (either open or reinfibulation); providers' confidence in identifying factors that are contraindications in performing deinfibulation and repair (either open or reinfibulation); understanding implications of FGM/C
Elliot et al. (2016)^30^	Pre‐ and post‐intervention group	Psychosexual therapists attending the College for Sex and Relationship Therapist (COSRT) 2‐day annual conference (*n* = 49). Setting: England	The 90‐min interactive group workshop with the aim to increase FGM‐related knowledge and skills in the participants. It deliberately moved away from didactic methods and favored skills acquisition through questions, comments, discussion, and debate. The workshop was repeated on four separate occasions during the 2‐day conference to maximize attendance at parallel conference events The intervention began with a small group exercise inviting diverse reactions and questions relating to FGM. This was followed by a brief slide presentation to the entire group, with pauses between the following segments for questions and reactions. It ended with a plenary discussion to draw out key learning points. A handout based on the teaching slides and a resource list including useful websites, training videos, books, and articles accompanied the workshop, and participants were encouraged to look further into the topic, discuss their learning with colleagues, and identify further needs for training and supervision	Knowledge of FGM types; providers' attitude towards FGM; understanding implications of FGM/C
Barnawi (2018)^31^	Quasi‐experimental quantitative approach with a non‐randomized, one‐group, pre‐/post‐test design.	Undergraduate nursing students who enrolled in the Bachelor's degree nursing program enrolled as full‐time students in Decker School of Nursing (DSON) at Binghamton University, USA (*n* = 86)	To examine the impact of the FGC digital e‐book on the level of attitude, knowledge, and self‐efficacy among the undergraduate nursing students. They had to watch the FGC digital e‐book and their knowledge was assessed before and after	Impact of the FGC digital e‐book (the intervention) on the level of attitude towards FGM among undergraduate nursing students; impact of the FGC digital e‐book on the level of perception/knowledge among undergraduate nursing students; impact of the FGC digital e‐book on the level of self‐efficacy among undergraduate nursing students
Kimani et al. (2018)^32^	Pre‐ and post‐ intervention study	Nurse‐midwives (*n* = 26). Setting: Kenya	Training was conducted using content on FGM/C extracted from the UNFPA training modules e‐tool for midwives for 3 days. Training sessions using interactive, trainer‐guided questions and group discussions through moderated plenaries were adopted. The session facilitators included researchers of diverse background relevant to FGM/C, notably Gynecologic/Obstetric, Nursing/Midwifery, Social Sciences, and Sexual Medicine affiliated to Africa Coordinating Centre for Abandonment of FGM/C (ACCAF). Training tool had in‐built electronic‐derived paper‐based pre‐ and post training quiz comprising 12 questions. The quiz assessed the following factors: definition, classification, determining factors, epidemiology, medicalization, prevention, health consequences, and nurse‐midwives' roles in FGM/C prevention themes	Knowledge of FGM types; knowledge of FGC immediate and long‐term complications; providers' confidence in patient counseling; confidence to practice deinfibulation and repair; perception about FGM; benefit of FGM; medicalization of FGM/C
Hess et al. (2022)^33^	A quasi‐experimental pre‐/post‐test study with a convenience sample (a control arm)	3rd‐year nursing students (*n* = 35). Setting: Nursing schools in the Midwestern USA	Students took a pre‐test, did a reading assignment and then attended a virtual, dramatization simulation session with a standardized patient; a Muslim woman with a personal history of FGC; then took the post‐test within the next week	Knowledge of FGM and its complications; understanding implications of FGM/C

Abbreviations: ACNM, American College of Nurse‐Midwives; ANC, antenatal care; CNM, certified nurse‐midwife; FGM/C, female genital mutilation/cutting; FGM, female genital mutilation; PCC, person‐centered communication; NGO, non‐governmental organization; RCT, randomized controlled trial; RH, reproductive health.

**TABLE 2:** Characteristics of excluded studies.

Study ID	Reason for exclusion
ACOG (2020)	A report
Adekanle (2011)	A survey (non‐intervention study)
Ali (2012)	A report
Ashimi (2014)	A survey (non‐intervention study)
Bankolé Sanni (1997)	Wrong study population
Calvert (2020)	A survey (non‐intervention study)
Chelala (1998)	Wrong study population
Deane (2022)	A survey (non‐intervention study)
Diop (2004)	Wrong study population
Diop (2008)	Wrong study population
El‐Gibaly (2019)	Non‐FGM intervention
Eyega (1997)	A survey (non‐intervention study)
Fay (2022)	A survey (non‐intervention study)
Galal (2022)	A survey (non‐intervention study)
Galukande (2015)	Wrong study population
Hess (2010)	A survey (non‐intervention study)
Jones (2023)	A survey (non‐intervention study)
Jorgensen (1998)	A report
Kangoum (1992)	Wrong study population
Kaplan (2013)	A survey (non‐intervention study)
Kaplan‐Marcus (2010)	A survey (non‐intervention study)
Kouta (2023)	A survey (non‐intervention study)
Leye (2006)	A report
Nybro (1998)	A case study
Refaat (2009)	A survey (non‐intervention study)
Relph (2013)	Non‐FGM intervention
Rogowska‐Szadkowska (2009)	A report
Tantet (2018)	A survey (non‐intervention study)
Thomas (2022)	A survey (non‐intervention study)
Thompson (2013)	A report
Ventura (2021)	A survey (non‐intervention study)
WHO (2001a)	Non‐FGM intervention
WHO (2001b)	A training guide
WHO (2006)	A survey (non‐intervention study)
WHO (2010)	A report
WHO (2011)	A report
WHO (2022a)	A training manual
WHO (2022b)	A training manual
Wyss (2022)	No control arm and not pre‐post design
Ziyada (2023)	A survey (non‐intervention study)

Abbreviation: FGM, female genital mutilation.

The included studies were carried out between 1998 and 2022. Five of the studies were noncontrolled pre‐ and post‐test studies,^17^, ^29^, ^30^, ^31^, ^32^ two were controlled non‐randomized studies,^28^, ^33^ and one was a cluster RCT.^34^ One study was carried out in Kenya, two in Mali, three in the United States, one in the United Kingdom, and one was a multicenter study carried out in Guinea, Kenya, and Somalia. Participants included different cadres of healthcare workers: obstetrician and gynecologist (*n* = 1), midwives (*n* = 2), psychosexual therapists (*n* = 1), nursing students (*n* = 2), and antenatal care (ANC) providers, which includes nurses, midwives, and other healthcare workers (*n* = 2). One study did not specify the cadre of healthcare worker, but targeted reproductive health service providers.

The interventions varied in delivery method, including in‐person presentation, lectures, group discussions, and hands‐on skills building.^17^, ^28^, ^30^, ^32^ One study used a self‐directed learning female genital cutting (FGC) digital e‐book^31^ and another conducted a virtual dramatization with a standardized patient who had a history of FGM.^33^ In‐person sessions typically combined videos and hands‐on techniques. One study incorporated diverse participatory adult learning methods, including value clarification exercises, storytelling, interactive games, demonstrations, and role‐plays.^34^ All interventions included theoretical content on FGM classification, complications, treatments, and prevalence. The duration of the intervention was in the range of 90 min to 2 months. The RCT compared a PCC training for FGM prevention with a health‐system strengthening approach; the control arm received only the latter.^34^

**FIGURE 2:**
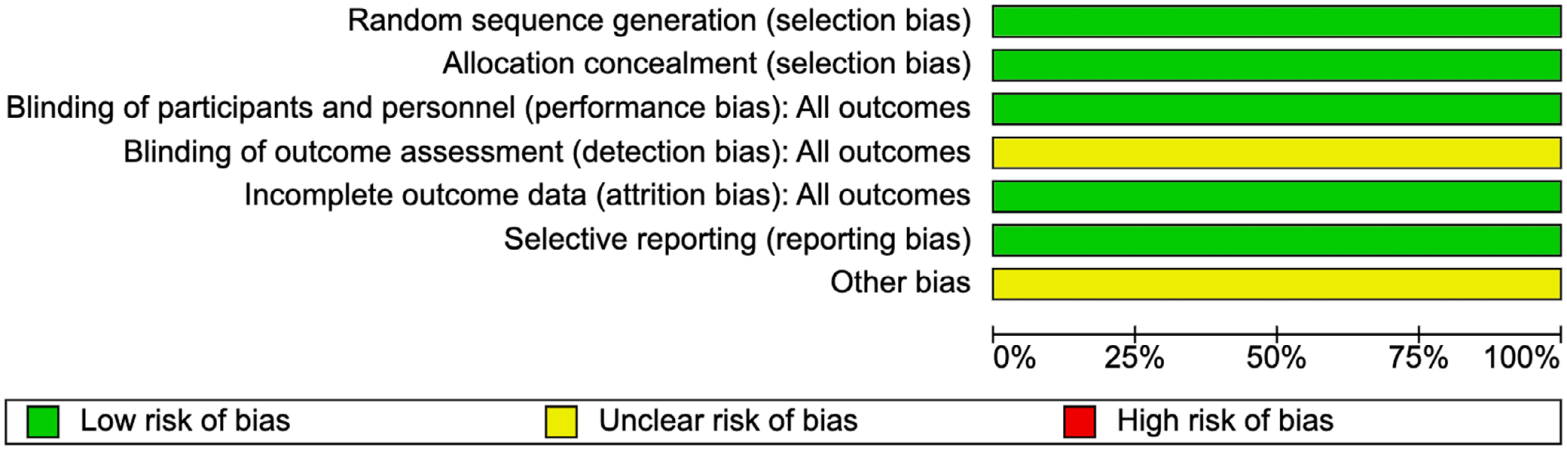
Risk of bias (Balde 2024). *Source*: Review Manager (RevMan) Web.

The Cochrane Risk of Bias tool for RCTs was used to assess the RCT, which was judged low risk in all domains except for detection bias for self‐reported outcomes, which was judged unclear as it was not reported in the article.^34^ In addition, the study was judged as unclear for other bias, as the authors reported possible selection bias in provider enrollment due to the use of clinical rotation schedules (Figure 2).

The risk of bias for the observational studies was assessed using the ROBINS‐I (Table 3).^25^ Overall, the studies were judged to have a “moderate” risk of bias. The studies were judged to be of moderate risk on the following domains: confounding,^28^, ^32^ selection bias,^28^ deviation from intended intervention,^31^ missing data,^28^ and outcome measurement.^32^ All the studies lacked a published protocol and were therefore judged as moderate for reporting bias. The use of interviewers posed a risk of interviewer bias, due to potential influence of preconceptions.^28^ Self‐report instruments are prone to biases, as participants may provide socially acceptable answers or may interpret the questions incorrectly. In one study, an electronic quiz allowed repeated attempts, but it was unclear if this applied to both pre‐ and post‐tests, possibly biasing reported outcomes.^32^ Two other studies used voluntary participant sampling to select participants, introducing potential sampling bias.^31^, ^33^

**TABLE 3:** Risk of Bias (ROBINS‐I tool): Non‐randomized control studies.

Study ID	Is confounding of the effect of intervention unlikely in this study?	Bias in selection of participants into the study	Bias in measurement of intervention	Bias due to departures from intended interventions	Bias due to missing data	Bias in measurement of outcomes	Bias in selection of the reported result	Overall bias	Did authors employ matching or restriction at design stage?	Did authors employ stratification or regression modeling in their analysis?
Diop et al. (1998)^28^ (same as Diop, 2007)	Moderate	Moderate	Low	Low	Moderate	Low	Moderate	Moderate	No	No
	Limited information to assess this domain	All health personnel (focused on OB‐GYN practitioners) were said to be included in the different sites. No detail on how the recruitment or selection of the sites was carried out	The different interventions were well defined	Intervention was implemented over a short period of time; switches were unlikely as experimental and control sites are in different communities	4 people were said to be missing during the analysis, but it was not specified if they were from the experimental or control sites; also, for some of the post‐test data, some of the questions were not completed by the participant	Method of outcome assessment was comparable across groups and not likely to be influenced by knowledge of the intervention received	Study did not report comparison between the intervention and control groups regarding some of their stated objectives, thus not free of selective reporting			
Hess et al. (2022)^33^	Low	Low	Low	Low	Low	Low	Moderate	Moderate	No	No
	Bias due to confounding is unlikely	Baseline characteristics were similar between groups. The same participant included in pre‐ and post‐surveys	The different interventions were well defined	No deviations from the intended intervention	The data were reasonably complete. One student did not complete the pre‐test, the other student did not complete the post‐test; both were in the control group	The method of outcome assessment was comparable across groups; researchers were blinded to the participants' completed tests	Unable to access this as we did not have access to protocol			
Newman and Nelson (2003)^29^	Low	Low	Low	Low	No information	Low	Moderate	Moderate	No	No
	No confounding expected	Study followed the same participants from pre‐intervention to post‐intervention, selection bias unlikely	Intervention status was well‐defined	Unlikely because the curriculum was a standardized video recording on FGM prevention	No information is reported about missing data or the potential for data to be missing	The method of outcome assessment was comparable at the before‐ and after survey	No information was provided to assess this domain			
Jacoby and Smith (2013)^17^	Low	Low	Low	Low	Low	Low	Moderate	Moderate	No	No
	No confounding expected	Study followed the same participants from pre‐intervention to post‐intervention, selection bias unlikely	Intervention status was well‐defined	No deviation from the intervention reported	All participants were analyzed	The method of outcome assessment was comparable at the before‐ and after survey	Unable to access this as we did not have access to protocol			
Elliot et al. (2016)^30^	Low	Low	Low	Low	Low	Low	Moderate	Moderate	No	No
	No confounding expected	Study followed the same participants from pre‐intervention to post‐intervention, selection bias unlikely	Intervention status was well‐defined	No deviation from the intervention reported	All participants were analyzed	The method of outcome assessment was comparable at the before‐ and after survey	Unable to access this as we did not have access to protocol			
Barnawi (2018)^31^	Low	Low	Low	Moderate	Moderate	Low	Moderate	Moderate		
	No confounding expected (all participants were in the same medical field)	Study followed the same participants from pre‐intervention to post‐intervention, selection bias unlikely	Intervention status was well‐defined.	The study used a FGC digital e‐book. The research relied on the students' skills of using audiovisual technology, which may vary from one student to another. Even though the author provided clear guidelines on how to utilize the FGC digital e‐book, some students may have skipped through some sections, not completing the e‐book content.	It was not clear how many students completed the pre‐test and how many completed the post‐test. The author mentioned 10 participants who dropped out of the post‐test after accessing the FGC digital e‐book. Reference was made to “the loss of study subjects due to the limited time and students' commitment to other coursework.”	The method of outcome assessment was comparable at the before‐and‐after survey.	Unable to access this as we did not have access to protocol.		No	No
Kimani et al. (2018)^32^	Moderate	Low	Low	Low	Low	Moderates	Moderate	Moderate	No	No
	Difference in the educational qualifications of participants: 19 had a diploma, two had a certificate, five participants had a Bachelors degree and two had a Masters degree	The same participants from pre‐intervention to post‐intervention, selection bias unlikely	Intervention status was well‐defined	No deviation from the intervention reported	All participants were analyzed	Study reported that “the tool had an inbuilt electronic‐supported quiz programmed in such a way that one must correctly answer the preceding questions before proceeding to the next. The tool enabled participants to retake questions until the correct response was achieved.” Unclear if this was pre‐ or post‐survey	Unable to access this as we did not have access to protocol			

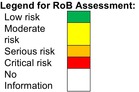

### 3.1 Effect of intervention

#### 3.1.1 Training of healthcare providers plus provision of IEC materials compared to provision of IEC materials only (RCT)

##### 3.1.1.1 Knowledge of FGM

Moderate‐quality evidence showed that ANC providers in the intervention arm had higher mean scores of FGM‐related knowledge (2.5, 95% Cl 2.2–2.7) compared to the control arm (1.6, 95% CI 1.5–1.8; *P* < 0.001).^34^

##### 3.1.1.2 Communication skills and confidence to provide FGM prevention and care to their clients

The intervention consisted of an interactive training to build ANC providers' knowledge of FGM using a component of PCC, where ANC providers were trained on a step‐by‐step approach to “Assess” the client's views on FGM, address and challenge her “Beliefs”, encourage “Change”, and together with the client, “Discuss and Decide” (ABCD).

##### 3.1.1.3 Providing appropriate FGM‐related prevention and care services

Moderate‐quality evidence showed that ANC providers in the intervention arm had a higher mean score of providing appropriate FGM prevention and care services (6.2, 95% Cl 5.9–6.6) compared to the providers in the control arm (3.7, 95% Cl 3.2–4.1) with a difference in the mean score of 2.5 (95% Cl 2.0–3.2; *P* < 0.001).^34^

In addition, clients reported that compared to ANC providers in the control group, those in the intervention group were more likely to implement components of PCC when addressing FGM prevention. Those in the intervention group were nearly nine times more likely to ask clients about FGM (adjusted odds ratio [AOR] 8.9, 95% CI 6.9–11.5; *P* < 0.001), nearly 10 times more likely to ask about clients' personal beliefs on FGM (AOR 9.7, 95% CI 7.5–12.5; *P* < 0.001), over nine times more likely to discuss the reasons for preventing FGM (AOR 9.2, 95% CI 7.1–11.9; *P* < 0.001), and nearly eight times more likely to talk about how FGM could be prevented (AOR 7.7, 95% CI 6.0–9.9; *P* < 0.001) compared to the ANC providers in the control group.^34^

##### 3.1.1.4 Confident in the knowledge to provide FGM prevention and care services

**FIGURE 3:**
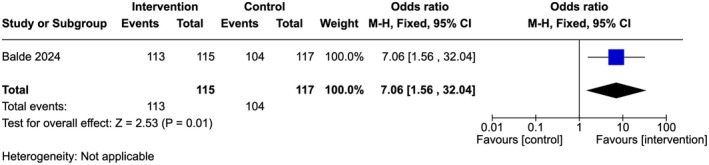
Confident in the knowledge to provide FGM prevention and care services.

Moderate‐quality evidence showed that ANC providers in the intervention arm had higher odds of being confident in their knowledge to provide FGM prevention and care services compared to those in the control arm (OR 7.06, 95% Cl 1.56–32.04; one study, 232 participants) (Figure 3).^34^

##### 3.1.1.5 Appropriate interpersonal communication skills

**FIGURE 4:**
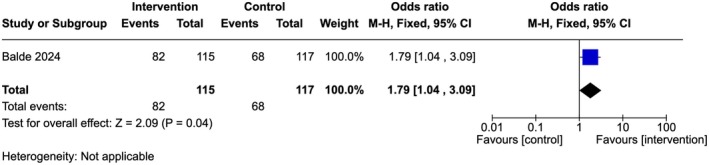
Appropriate interpersonal communication skills.

Moderate‐quality evidence showed that there was no statistically significant difference between the number of healthcare providers in the intervention arm compared to the control arm regarding appropriate interpersonal communication skills (OR 1.79, 95% Cl 1.04–3.09; one study, 232 participants) (Figure 4).^34^

##### 3.1.1.6 Change in attitude about support for FGM, FGM medicalization, and reinfibulation

**FIGURE 5:**
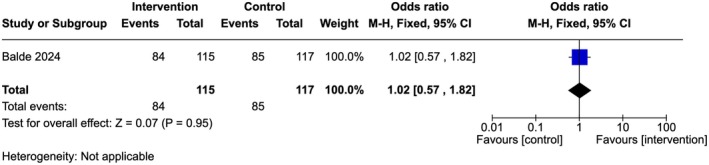
Less supportive attitude towards FGM.

**FIGURE 6:**
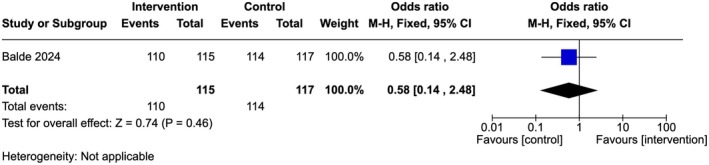
Not supportive of FGM.

**FIGURE 7:**
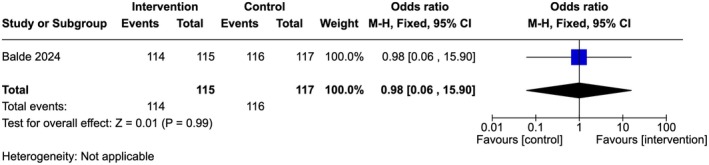
Not supportive of medicalized FGM.

Low‐quality evidence showed that there is no significant difference between the groups (OR 1.02, 95% Cl 0.57–1.82; one study, 232 participants) (Figure 5). A similar trend was observed among the providers in both groups reporting that they did not support FGM (96% in the intervention arm compared to 97% in the control arm [OR 0.58, 95% Cl 0.14–2.48; one study, 232 participants]) (Figure 6) and/or medicalized FGM (99% in both arms [OR 0.98, 95% Cl 0.06–15.9; one study, 232 participants]) (Figure 7).^34^

#### 3.1.2 Educational intervention (face‐to‐face, group discussion, hands‐on demonstration) compared to no intervention control arm (non‐randomized)

##### 3.1.2.1 Knowledge of FGM

**FIGURE 8:**
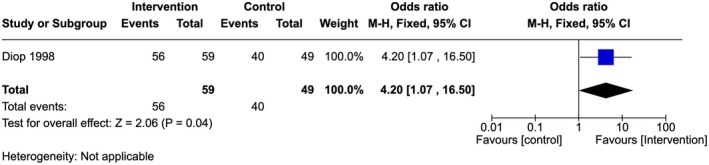
Knowledge of the different types of FGM.

Very low‐quality evidence showed that there was a significant difference between the number of providers that could recognize the different types of FGM in the intervention compared to the control arm. Providers in the intervention arm had higher odds of recognizing the different types of FGM compared to providers in the control sites (OR 4.20, 95% CI 1.07–16.50; one study, 108 participants) (Figure 8).^28^

**FIGURE 9:**
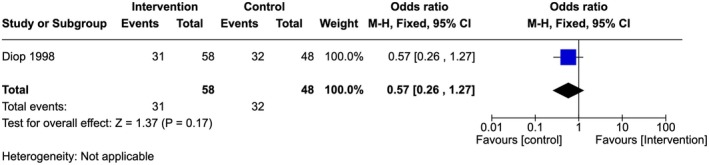
Number of providers who disagree that FGM posed no health risk in a hygienic environment.

Very low‐quality evidence showed that when asked whether FGM posed no health risk when carried out in a hygienic environment; there was no statistically significant difference between the providers (54%) in the intervention site compared to 66% of providers in the control sites who disagreed (OR 0.57, 95% CI 0.26–1.27; one study, 106 participants) (Figure 9).^28^

##### 3.1.2.2 Ability to identify FGM‐related complications

One study reported similar increase in the number of providers in both arms that could mention at least three immediate complications. In the intervention arm, the number of providers increased from 40% to 86%, and knowledge of subsequent complications increased from 49% to 72% at follow‐up. To a lesser degree, respondents from the control sites also registered improvement in their knowledge of FGM‐related complications, with an increase from 61% to 73% at follow‐up.^28^ The quality of evidence was very low.

##### 3.1.2.3 Communication skills and confidence to provide FGM prevention and care

One study reported that a significant majority (96%) of the healthcare providers who used the flip chart said it helped them communicate messages more effectively. However, some found it constraining, while 38% felt it did not limit their ability to convey information. Among the 63% who thought they were constraining, 56% still found it useful.^28^ It was not specified how the result applied to the experimental and control sites.

The study also reported that more than half of the health agents who had received IEC training were unwilling to address FGM‐related issues with clients compared to one‐third of those who had not been trained in communication techniques.^28^

##### 3.1.2.4 Provider view of their active role in anti‐FGM activities

**FIGURE 10:**
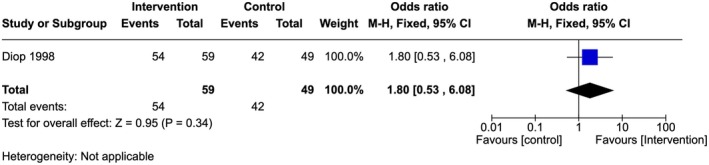
Provider view of their active role in anti‐FGM activities.

There was no statistically significant difference in the number of providers willing to conduct IEC activities in the intervention sites (91.5%) compared to the control sites (85.7%) (OR 1.80, 95% Cl 0.53–6.08; one study, 108 participants) (Figure 10).^28^ The quality of evidence was very low.

#### 3.1.3 Virtual, dramatization simulation session with a standardized patient with FGM compared to no intervention control arm (non‐randomized)

##### 3.1.3.1 Knowledge of FGM

Very low‐quality evidence showed that training improved knowledge of FGM with a mean score of 9.50 ± 1.345 in the intervention group compared to 6.76 ± 4.640 in the control group (mean difference [MD] 2.74, range 0.59–4.89).^33^

##### 3.1.3.2 Knowledge of the consequences of FGM

**FIGURE 11:**
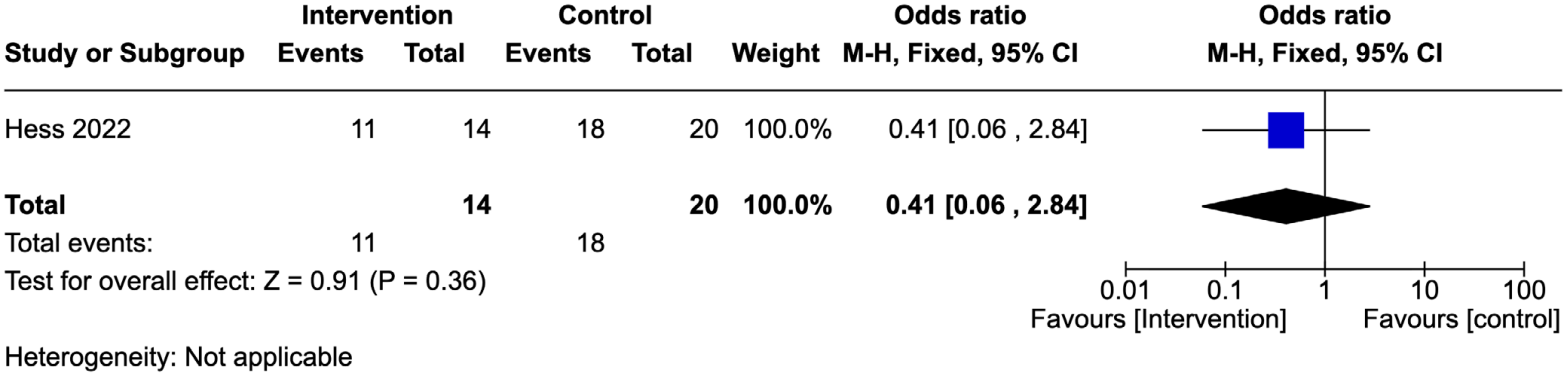
Knowledge on the consequences of FGM.

Very low‐quality evidence showed that there was no difference in the number of providers in both arms regarding the knowledge that the consequences of FGM are numerous (OR 0.41, 95% Cl 0.06–2.84; one study, 34 participants) (Figure 11).^33^

##### 3.1.3.3 Change in attitude about support for FGM, FGM medicalization, and reinfibulation

One study reported that more participants thought FGM should not be medicalized (26.5% increase) on the post‐test.^33^ No information was provided for the different arms in both studies and the quality of evidence was very low.

#### 3.1.4 Educational intervention (face‐to‐face, group discussion, hands‐on demonstration) (pre‐ and post‐test study)

##### 3.1.4.1 Knowledge of FGM

All studies that assessed the outcome reported an improvement in knowledge after the intervention. One study reported an increase from a mean of 2.36 to 4.18.^17^ Another study reported that the number of participants who knew the number of the types of FGM increased from 35% to 85% (*χ* ^2^ [6, *N* = 97] = 29.10; *P* < 0.001).^30^ One study reported a significant improvement in participants' knowledge after a 3‐day training, from 64% at baseline versus 96.2% after training (*t*(25) = 7.408; *P* < 0.001), and knowledge of the types of FGM/C, from 84.6% at baseline versus 100% after training (*t*(25) = 2.132; *P* = 0.043).^32^ The quality of evidence for all the studies was very low.

##### 3.1.4.2 Confidence and ability to identify FGM‐related complications

After training, one study reported improved scores in participants' knowledge of FGM‐related health consequences (immediate physical, *P* = 0.003; gynecological, *P* = 0.001; obstetric, *P* = 0.022; sexual, *P* < 0.001; and social harms, *P* < 0.001). There was no statistically significant difference in knowledge scores regarding FGM‐related psychological complication after intervention.^32^ The quality of evidence for all the studies was very low.

##### 3.1.4.3 Change in attitude about support for FGM, FGM medicalization, and reinfibulation

One study found no significant difference before and after the intervention.^30^ In another study, all the participants agreed that FGM/C violates women's and girls' human rights (*P* = 0.043) and that it was not safer if FGM was performed by healthcare workers.^32^ The study further assessed the capacity of participants to resist reinfibulation if requested by a woman supposedly under pressure from her husband and reported significant improvement on the need for the woman to remain deinfibulated (*P* < 0.001).^32^ Participants' knowledge on the importance of the need to leave the woman deinfibulated was reported to be poor to moderate.^32^ There was also an improvement after training with providers pledging to never perform FGM because it is not medically necessary and causes harm (*t*(25) = −5.000; *P* < 0.001). The quality of evidence for all the studies was very low.

##### 3.1.4.4 Communication skills and confidence to provide FGM prevention and care to their clients

One study reported an increase in the mean confidence level from 2.36 to 4.09 among healthcare workers regarding counseling women with type III FGM. The mean confidence level of healthcare provider increased from 1.54 to 3.54 after training on deinfibulation and repair using simulated pelvic models.^17^ The quality of evidence for all the studies was very low.

#### 3.1.5 Virtual training only (before‐and‐after study)

##### 3.1.5.1 Knowledge of FGM

One study reported that an FGM digital e‐book significantly improved the attitudes of nursing students on FGM, with an increase in mean FGM knowledge scores from 32.95 ± 2.977 at baseline to 38.49 ± 2.895 after the intervention (MD 5.54 ± 4.5, range − 6.495 to −4.583; *P* < 0.001).^31^ The quality of evidence for all the studies was very low.

##### 3.1.5.2 Communication skills and confidence to provide FGM prevention and care to their clients

In one study that provided virtual training, after the intervention, providers were three times more likely to ask pregnant women if they have FGC complications that might affect birthing.^29^ Another study reported an increase in the level of readiness of students to provide competent care to manage FGM from a mean of 15.7629 ± 1.88 at baseline to 18.567 ± 1.89 after the intervention (MD –2.80, range − 3.37 to −2.24; *P* < 0.001).^31^ The quality of evidence for all the studies was very low.

##### 3.1.5.3 Management of patients with FGM‐related complications

One study assessed the number of FGM‐related complications that were treated on site and reported that the mean number of such FGM‐related complications increased by 26%.^29^ The quality of evidence was very low.

##### 3.1.5.4 Change in attitude about support for FGM, FGM medicalization, and reinfibulation

One study reported a significant improvement regarding the attitude toward FGM among nursing students after training. The mean FGM attitude scores increased from 49.42 ± 5.475 at baseline to 54.89 ± 5.902 after the intervention (MD 5.47, SD 8.3, range − 7.18 to −3.77; *P* < 0.001).^31^ The quality of evidence was very low.

##### 3.1.5.5 Skills on providing FGM‐related education and counseling for communities in outreach services

One study reported an improvement in healthcare workers providing counseling on FGM, which was nonexistent at baseline. The study reported that 414 women receiving antenatal care also received counseling on abandoning FGM. In addition, nearly 75% of providers assessed passed the counseling skill performance test. The study also showed that healthcare workers were present as resource persons at 714 of 1187 community outreach sessions and 473 educational sessions where they discussed the negative effects of FGM.^29^

None of the included studies reported on the reduction of FGM‐related stigma, discrimination, and disrespectful care or the recording of FGM in clinical reports.

## 4 DISCUSSION

This review sought to determine the impact of FGM education on healthcare providers' knowledge, attitudes, skills, and quality of care for girls and women with FGM. The results showed that the training, whether delivered face‐to‐face or online, improved healthcare workers' knowledge of FGM and its complications. Healthcare workers reported increased confidence in identifying and managing FGM‐related complications, enhanced communication and counseling skills, with some improvement in attitudes toward FGM, its medicalization, and reinfibulation. Some training institutions offer materials such as job aids and handbooks on FGM; however, gaps remain in many countries. Findings from this review indicate that the provision of these materials alone is insufficient to enhance knowledge; however, emphasis should also be placed on capacity building, which involves teaching healthcare workers how to effectively utilize these resources and to build skills and competencies. One study demonstrated a significant increase in resource use of 91% among the intervention group compared to 56% in the control group, who also had access to the resources but did not receive additional training.^34^ This highlights the importance of capacity building for healthcare workers, suggesting that training and ongoing support play a key role. The same study reported improved healthcare workers' attitudes, making them less supportive of the practice.^34^

In‐depth interviews with healthcare workers and ANC clients revealed that healthcare workers found the ANC context to be a feasible, acceptable, and appropriate entry point for FGM prevention counseling. Likewise, clients were satisfied with the communication received and trusted their ANC provider as a source of information. Healthcare workers also suggested integrating such discussions at other service delivery points within health facilities and involving different cadres of healthcare workers (e.g., community healthcare workers) to increase the sustainability and impact of the intervention.^35^

FGM training programs generally enhance theoretical knowledge and confidence, often focusing on classification, complications, and counseling over practical, hands‐on clinical skills.^36^, ^37^ This may create a false sense of diagnostic competence, particularly in legal settings where misclassification carries serious consequences.^38^, ^39^ Research shows that aside from infibulation, most forms of FGM are difficult to identify, even for experienced clinicians, challenging the assumption that FGM types can be reliably and visibly distinguished through theoretical training alone.^40^

The included studies on FGM training were conducted in diverse locations, encompassing both low‐ and high‐income countries, suggesting the effectiveness of implementing such education in different settings. However, the varied prevalence of FGM in these countries limits the broad generalization of findings. The mode and duration of intervention delivery varied across studies, ranging from face‐to‐face sessions to virtual simulations lasting 35 min to 4 days. Despite these differences, improvements in reported outcomes were consistent, indicating that the mode of intervention might not significantly impact the results. Most studies had short follow‐up periods, typically concluding at the end of the intervention, making it difficult to reliably assess long‐term changes in knowledge and attitude. Longer follow‐up studies, extending to 1 year or longer, are needed to better understand lasting impacts. In some non‐randomized controlled studies, both the intervention and control groups showed similar improvements in certain outcomes, raising questions about whether changes can be solely attributed to the intervention.^28^, ^33^ Participants in the control group may have independently sought information on FGM after the baseline assessments.

Evidence from some studies suggest that although training can improve awareness, it may lead to unintended consequences, such as stigmatization or excessive questioning of women with FGM, potentially resulting in poorer care experiences.^41^, ^42^ Although these outcomes were beyond the scope of this review, they underscore the importance of sensitive, person‐centered approaches. One of the included studies reported that PCC improved ANC providers' engagement on FGM prevention. Providers in the intervention group were 9–10 times more likely to initiate discussions on FGM‐related topics, including beliefs, abandonment, and prevention. These findings suggest that capacity building can strengthen both provider knowledge and respectful, effective communication.^34^

Notably, none of the studies evaluated the impact of FGM training on local health practitioners, such as traditional birth attendants and community healthcare workers who play a role in FGM practices in some settings and can be transformed into advocates against the practice given their proximity to affected communities. Consequently, the influence of these interventions on the knowledge and attitudes of such practitioners remains unclear.

This review aligns with previous research suggesting that FGM‐related training improves provider knowledge and attitudes [43]. It also supports recommendations to include such training during both pre‐licensing and in‐service education for health professionals.^43^, ^44^

### 4.1 Strength and limitations

The review utilized a comprehensive search of relevant databases without restrictions in study type or language. Following Cochrane methodology, it employed a robust approach, including reviewer blinding and a validated screening tool to minimize bias.^22^, ^24^ Despite this, few studies met the inclusion criteria, with only one RCT. Some studies had small sample sizes, which limited the ability to determine significance.^17^, ^29^, ^30^, ^33^ Variation in intervention delivery and duration prevented meta‐analysis. No study assessed the long‐term effects on information retention or changes in providers' attitudes. Only two studies assessed client satisfaction with health services after the intervention.^28^, ^34^ Finally, outcomes relied on self‐reports or interviews, potentially introducing self‐report bias.

## 5 CONCLUSION

This review indicates that training influences healthcare workers' knowledge and attitudes toward FGM. However, the limited number of studies and lack of high‐quality evidence prevent definitive conclusions about the effectiveness of educational interventions or the best ways to implement them. These limitations reduce the reliability and generalizability of the findings. More rigorous, multicentered studies are needed, especially in FGM‐prevalent regions and industrialized countries with large immigrant populations. Future research should consider regional, ethnic, and sociodemographic differences with long‐term follow‐up to assess sustained changes in provider knowledge and attitudes.

## AUTHOR CONTRIBUTIONS

CO and SO coordinated the screening and data extraction, performed the data analysis, interpreted the results, and drafted the manuscript. AO contributed to the screening, data extraction, and analysis and drafting of the manuscript. MO and FO contributed to the screening and drafting of the manuscript. ON participated in interpretation of the results and writing of the manuscript. CCP contributed to the development of the research question and drafting of the manuscript. MM conceptualized the study, coordinated its planning and implementation, and supervised the analysis of the data and writing of the manuscript. All authors commented on and approved the final manuscript.

## FUNDING INFORMATION

This work received funding from the Government of Norway and the UNDP‐UNFPA‐UNICEF‐WHO‐World Bank Special Programme of Research, Development, and Research Training in Human Reproduction (HRP), a cosponsored programme executed by the World Health Organization (WHO).

## CONFLICT OF INTEREST STATEMENT

The authors have no conflicts of interest.

## Supporting information


**Table S1:** Search strategy.

**Table S2:** **a‐e:** GRADE tables.

## Data Availability

Research data are not shared.

## References

[1] World Health Organization, United Nations Population Fund & United Nations Children's Fund (UNICEF) . Female genital mutilation: a joint WHO/UNICEF/UNFPA statement. World Health Organization; 1997 [WHO website]. Accessed May 14, 2023. https://iris.who.int/handle/10665/41903

[2] United Nations Children's Fund . Female Genital Mutilation: A global concern. 2024 Update. UNICEF; 2024 Accessed April 3, 2025. https://data.unicef.org/resources/female‐genital‐mutilation‐a‐global‐concern‐2024/

[3] World Health Organization . Female genital mutilation. World Health Organization (WHO). January 31, 2025. Accessed April 5, 2025. https://www.who.int/news‐room/fact‐sheets/detail/female‐genital‐mutilation

[4] Yoder PS , Abderrahim N , Zhuzhuni A . Female Genital Cutting in the Demographic and Health Surveys: A Critical and Comparative Analysis. DHS Comparative Reports No. 7. ORC Macro; 2004.

[5] World Health Organization . Female Genital Mutilation. Integrating the Prevention and the Management of the Health Complications into the curricula of nursing and midwifery: Teachers Guide. 2001 [WHO website]. Accessed May 16, 2023. https://www.who.int/publications/i/item/WHO‐RHR‐01.16

[6] Chalmers B , Omer‐Hashi K . What Somali women say about giving birth in Canada. J Reprod Infant Psychol. 2002;20:267‐282.

[7] Doucet MH , Pallitto C , Groleau D . Understanding the motivations of health‐care providers in performing female genital mutilation: an integrative review of the literature. Reprod Health. 2017;14(1):46. 28335771 10.1186/s12978-017-0306-5 PMC5364567

[8] Balde MD , O'Neill S , Sall AO , et al. Attitudes of health care providers regarding female genital mutilation and its medicalization in Guinea. PLoS One. 2021;16(5):e0249998. 33983949 10.1371/journal.pone.0249998 PMC8118326

[9] Lurie JM , Weidman A , Huynh S , Delgado D , Easthausen I , Kaur G . Painful gynecologic and obstetric complications of female genital mutilation/cutting: a systematic review and meta‐analysis. PLoS Med. 2020;17(3):e1003088. 32231359 10.1371/journal.pmed.1003088 PMC7108709

[10] Gebremicheal K , Alemseged F , Ewunetu H , et al. Sequela of female genital mutilation on birth outcomes in Jijiga town, Ethiopian Somali region: a prospective cohort study. BMC Pregnancy Childbirth. 2018;18:305. 30029634 10.1186/s12884-018-1937-4 PMC6053719

[11] Vangen S , Stollenberg C , Johansen REB , Sundby J , Stray‐Perderson B . Perinatal complications among ethnic Somalis in Norway. Acta Obstet Gynecol Scand. 2002;81:317‐322. 11952461 10.1034/j.1600-0412.2002.810407.x

[12] Widmarck C , Tishelman C , Ahlberg BM . A study of Swedish midwives' encounters with infibulated African women in Sweden. Midwifery. 2002;1:113‐125. 10.1054/midw.2002.0307 12139909

[13] Johansen REB . Care for infibulated women giving birth in Norway: an anthropological analysis of health workers' management of a medically and culturally unfamiliar issue. Med Anthropol Q. 2006;20:516‐544. 17225657 10.1525/maq.2006.20.4.516

[14] Abdulcadir J , Rodriguez MI , Say L . Research gaps in the Care of Women with female genital mutilation: an analysis. BJOG. 2015;122(3):294‐303. 25514892 10.1111/1471-0528.13217

[15] Ahmed W , Gebretsadik E , Gbenou D , et al. Lessons learnt in scaling up evidence‐based comprehensive health sector responses addressing female genital mutilation in highly prevalent settings. BMJ Glob Health. 2023;8:e012270. doi:10.1136/bmjgh-2023-012270 PMC10277070 37308264

[16] Sangaré M , Tandia F , Touré K . Study of the effectiveness of training Malian Social and health agents in female genital cutting issues and in educating their clients. Population Council; 1998.

[17] Jacoby SD , Smith A . Increasing certified nurse‐midwives' confidence in managing the obstetric care of women with female genital mutilation/cutting. J Midwifery Womens Health. 2013;58(4):451‐456. 23931662 10.1111/j.1542-2011.2012.00262.x

[18] Nordmann K , Subirón‐Valera AB , King M , Küpper T , Martínez‐Pérez GZ . Management of Female Genital Mutilation/cutting‐related obstetric complications: a training evaluation. Int J Environ Res Public Health. 2022;19(15):9209. 35954566 10.3390/ijerph19159209 PMC9367947

[19] World Health Organization . Person‐centred communication for female genital mutilation prevention: a facilitator's guide for training health‐care providers. World Health Organization; 2022a. Licence: CC BY‐NC‐SA 3.0 IGO.

[20] World Health Organization . Integrating female genital mutilation content into nursing and midwifery curricula: a practical guide. World Health Organization; 2022b Licence: CC BY‐NC‐SA 3.0 IGO.

[21] Amasanti ML , Imcha M , Momoh C . Compassionate and proactive interventions by health Workers in the United Kingdom: a better approach to prevent and respond to female genital mutilation? PLoS Med. 2016;13(3):e1001982. 27002322 10.1371/journal.pmed.1001982 PMC4803291

[22] Higgins JP , Thomas J , Chandler J , et al. Cochrane Handbook for Systematic Reviews of Interventions. John Wiley & Sons; 2019. 10.1002/14651858.ED000142 PMC10284251 31643080

[23] Page MJ , McKenzie JE , Bossuyt PM , et al. The PRISMA 2020 statement: an updated guideline for reporting systematic reviews. BMJ. 2021;372:n71. 33782057 10.1136/bmj.n71 PMC8005924

[24] Covidence systematic review software, Veritas Health Innovation, Melbourne, Australia. Available at www.covidence.org

[25] Sterne JA , Hernán MA , Reeves BC , et al. ROBINS‐I: a tool for assessing risk of bias in non‐randomised studies of interventions. BMJ. 2016;355:i4919. doi:10.1136/bmj.i4919 27733354 10.1136/bmj.i4919 PMC5062054

[26] Review Manager (RevMan) [Computer program]. Version 7.2.0. The Cochrane Collaboration; 2024 https://revman.cochrane.org

[27] Campbell M , McKenzie JE , Sowden A , et al. Synthesis without meta‐analysis (SWiM) in systematic reviews: reporting guideline. BMJ. 2020;368:l6890. 31948937 10.1136/bmj.l6890 PMC7190266

[28] Diop NJ , Traore´ F , Diallo H . Study of the effectiveness of training Malian social and health agents in female genital cutting issues and in educating their clients. Population Council; 1998.

[29] Newman C , Nelson D . Mali: FGC. Counseling and advocacy to abandon female genital cutting. Training providers, reaching communities, Chapel Hill, North Carolina, IntraHealth International, PRIME. 2003.

[30] Elliott C , Creighton SM , Barker M , Liao L . A brief interactive training for health care professionals working with people affected by “female genital mutilation”: initial pilot evaluation with psychosexual therapists. Sex Relat Ther. 2016;31(1):70‐82.

[31] Barnawi N . The effects of a digital educational intervention on undergraduate nursing students' attitudes, knowledge and self‐efficacy with female genital cutting. Graduate Dissertations and Theses. 48. 2018.

[32] Kimani S , Esho T , Kimani V , et al. Female genital mutilation/cutting innovative training approach for nurse‐midwives in high prevalent settings. Obstet Gynecol Int. 2018:5043512. doi:10.1155/2018/5043512 29736171 10.1155/2018/5043512 PMC5875060

[33] Hess RF , Ross R , Wyss L , Donnenwirth JA . Nursing students' knowledge gained about female genital cutting/mutilation through dramatization simulation with a standardized patient: a quasi‐experimental study. Nurse Educ Today. 2021;116:105443. 10.1016/j.nedt.2022.105443 35717812

[34] Balde MD , Ndavi PM , Mochache V , et al. Cluster randomised trial of a health system strengthening approach applying person‐centred communication for the prevention of female genital mutilation in Guinea, Kenya and Somalia. BMJ Open. 2024;14:e078771. doi:10.1136/bmjopen-2023-078771 PMC11227771 38964796

[35] Ndavi PM , Balde MD , Milford C , et al. The feasibility, acceptability and appropriateness of implementing person‐centered communication for FGM prevention in antenatal care settings in Guinea, Kenya and Somalia. Glob Public Health. 2024;19(1):2369100. doi:10.1080/17441692.2024.2369100 38987991

[36] Abdulcadir J , Catania L , Hindin MJ , Say L , Petignat P , Abdulcadir O . Female genital mutilation. Obstet Gynecol. 2016;128(5):958‐963. 27741194 10.1097/AOG.0000000000001686

[37] Abdulcadir J , Dugerdil A , Boulvain M , et al. Missed opportunities for diagnosis of female genital mutilation. Int J Gynaecol Obstet. 2014;125(3):256‐260. 24713414 10.1016/j.ijgo.2013.11.016

[38] Johnsdotter S . Meaning well while doing harm: compulsory genital examinations in Swedish African girls. Sexual Reprod Health Matt. 2019;27(2):87‐99. doi:10.1080/26410397.2019.1586817 PMC7887926 31533584

[39] Johnsdotter S , Wendel L , Grönvall K , Essén B . Genital examinations in cases of suspected ‘female genital mutilation’ in Sweden 1982–2022: lawful decisions resulting in structural injustice. Humanit Soc Sci Commun. 2025;12(1): 1‐14 doi:10.1057/s41599-025-05476-6

[40] Berg RC , Underland V . Effectiveness of interventions designed to reduce the prevalence of female genital mutilation/cutting. Report from the Norwegian Knowledge Centre for the Health Services. 2014. 29320117

[41] Ali S , Karlsen S , Learner H , et al. UK policy response to female genital mutilation needs urgent rethink. Br Med J. 2023;383:e074751. 37996115 10.1136/bmj-2022-074751

[42] Karlsen S , Carver N , Mogilnicka M , Pantazis C . 'Putting salt on the wound': a qualitative study of the impact of FGM‐safeguarding in healthcare settings on people with a British Somali heritage living in Bristol, UK. BMJ Open. 2020;10(6):e035039. doi:10.1136/bmjopen-2019-035039 PMC7304797 32554738

[43] Dawson AJ , Turkmani S , Varon N , Nanayakkara S , Sulivan E , Homer CS . Midwives' experiences of caring for women with female genital mutilations: insights and ways forward for practice in Australia. Women Birth. 2015;18(3):207‐214. 10.1016/j.wombi.2015.01.007 25686876

[44] Evans C , Tweheyo R , McGarry J , et al. Crossing cultural divides: a qualitative systematic review of factors influencing the provision of healthcare related to female genital mutilation from the perspective of health professionals. PLoS One. 2019;14(3):e0211829. 30830904 10.1371/journal.pone.0211829 PMC6398829

