# Supplementary material for: Capacity building for healthcare workers on preventing and managing female genital mutilation: Impact on knowledge, attitudes, skills, and quality of care—A systematic review
Source: Int J Gynaecol Obstet. 2026 Jan 26;172(Suppl 1):48–64. doi: 10.1002/ijgo.70757 (PMC12833632; doi:10.1002/ijgo.70757)
Supplement: Supplementary file 1 — Table S1: Search strategy. [file IJGO-172-48-s002.docx]

# Table S1: Search Strategy

**CINAHL**

| S48 | S9 AND S47 |
| --- | --- |
| S47 | S41 NOT S46 |
| S46 | S44 NOT S45 |
| S45 | MH Human |
| S44 | S42 OR S43 |
| S43 | TI animal model* |
| S42 | MH animals+ OR MH (animal studies) |
| S41 | S25 OR S39 OR S40 |
| S40 | TI (case W1 (report OR reports)) OR AB (case W1 (report OR reports)) |
| S39 | S26 OR S27 OR S28 OR S29 OR S30 OR S31 OR S32 OR S33 OR S34 OR S35 OR S36 OR S37 OR S38 |
| S38 | "4 arm" OR "four arm" |
| S37 | (single OR doubl* OR tripl* OR treb*) W3 (blind* OR mask*) |
| S36 | AB ((strategy OR strategies) N3 (improv* OR education*)) |
| S35 | TI ((strategy OR strategies) N3 (improv* OR education*)) |
| S34 | AB ("control year" OR "experimental year" OR "control period" OR "experimental period") |
| S33 | TI ("control year" OR "experimental year" OR "control period" OR "experimental period") |
| S32 | AB (control N3 (area OR cohort OR compare* OR condition OR design OR group OR intervention OR participant OR study)) |
| S31 | AB ("time points" N3 (over OR multiple OR three OR four OR five OR six OR seven OR eight OR nine OR ten OR eleven OR twelve OR month OR hour OR day OR "more than")) |
| S30 | AB ((clinical OR epidemiologic OR evaluation OR validation) W2 (study OR studies OR trial)) |
| S29 | TI ((clinical OR epidemiologic OR evaluation OR validation) W2 (study OR studies OR trial)) |
| S28 | TI ( case* W3 (comparison* OR control* OR series) ) OR AB ( case* W3 (comparison* OR control* OR series) ) |
| S27 | AB (("before and after" OR case* OR cohort OR comparative OR controlled OR "cross section*" OR "follow up" OR longitudinal OR multicenter OR observation* OR prospective OR quasicontrol* OR quasi-control* OR quasiexperiment* or quasi-experiment* OR quasirandom* OR "record linkage" OR retrospective OR "time series") W3 (analy* OR design OR evaluat* OR investigat* OR method OR procedure OR study OR studies OR trial)) |
| S26 | TI (("before and after" OR case* OR cohort OR comparative OR controlled OR "cross section*" OR "follow up" OR longitudinal OR multicenter OR observation* OR prospective OR quasicontrol* OR quasi-control* OR quasiexperiment* or quasi-experiment* OR quasirandom* OR "record linkage" OR retrospective OR "time series") W3 (analy* OR design OR evaluat* OR investigat* OR method OR procedure OR study OR studies OR trial)) |
| S25 | S10 OR S11 OR S12 OR S13 OR S14 OR S15 OR S16 OR S17 OR S18 OR S19 OR S20 OR S21 OR S22 OR S23 OR S24 |
| S24 | AB cluster W3 RCT |
| S23 | MH crossover design OR MH comparative studies |
| S22 | AB control W5 group |
| S21 | PT randomized controlled trial |
| S20 | MH placebos |
| S19 | MH sample size AND AB (assigned OR allocated OR control ) |
| S18 | TI trial |
| S17 | AB random* |
| S16 | TI randomised OR randomized |
| S15 | MH cluster sample |
| S14 | MH pretest‐posttest design |
| S13 | MH random assignment |
| S12 | MH single‐blind studies |
| S11 | MH double‐blind studies |
| S10 | MH randomized controlled trials |
| S9 | S5 AND S8 |
| S8 | S6 OR S7 |
| S7 | (MH "Female Genital Mutilation") |
| S6 | (female or girl or girls or women or woman) N3 (circumcis* or "genital cut*" or "genital mutilat*") |
| S5 | S1 OR S2 OR S3 OR S4 |
| S4 | inform* or educat* or counsel* or communicati* or promoti* or program or campaign or initiative or attitud* or knowledge* |
| S3 | (MH "Attitude+") OR (MH "Counseling+") |
| S2 | (MH "Health Knowledge") OR (MH "Professional Knowledge+") OR (MH "Communication+") OR (MH "Health Promotion") |
| S1 | (MH "Health Education+") OR (MH "Patient Education+") OR (MH "Sex Education") |

**IRIS**

female circumcision and Subject: contains education

some duplicates and irrelevant removed manually

**Medline**

1. exp Health Education/ or exp health communication/ or exp health promotion/ or exp Health Knowledge, Attitudes, Practice/ or exp Counseling/

2. exp Health Personnel/ed [Education]

3. exp "Attitude of Health Personnel"/

4. (inform* or educat* or counsel* or communicati* or promoti* or program or campaign or initiative or attitud* or knowledge*).mp.

5. or/1-4

6. exp Circumcision, Female/

7. ((female or girl or girls or women or woman) adj3 (circumcis* or genital cut* or genital mutilat*)).mp.

8. 6 or 7

9. 5 and 8

10. (randomi?ed or placebo or randomly).mp.

11. clinical trials as topic.sh.

12. trial.ti.

13. 10 or 11 or 12

14. exp clinical trial/ or exp observational study/ or exp comparative study/ or exp evaluation study/ or exp multicenter study/ or exp validation study/

15. ((clinical or comparative or controlled or evaluation or multicenter or multi-center or multicentre or multi-centre or validation) adj3 (study or studies or trial?)).mp.

16. epidemiologic studies/ or exp case-control studies/ or exp cohort studies/ or exp controlled before-after studies/ or exp cross-sectional studies/ or exp historically controlled study/ or exp interrupted time series analysis/

17. (cohort$ or (case$ adj3 (comparison$ or control$ or series))).mp.

18. epidemiologic methods/

19. limit 18 to yr=1966-1989

20. (("before and after" or "before-and-after" or case$ or cross?section$ or "cross section$" or "follow up" or "follow-up" or longitudinal or observation$ or prospective or "record-linkage" or "record linkage" or retrospective or "time-series" or "time series") adj3 (analy$ or design or evaluat$ or investigat$ or method or procedure or study or studies or trial?)).mp.

21. ("quasi-experiment$" or quasiexperiment$ or "quasi experiment$" or "quasi random$" or "quasi-random$" or quasirandom$ or "quasi control$" or "quasi-control$" or quasicontrol$).mp.

22. (time points adj3 (over or multiple or three or four or five or six or seven or eight or nine or ten or eleven or twelve or month$ or hour? or day? or "more than")).ab.

23. (control$ adj4 (analy$ or area or cohort? or compar$ or condition or design or evaluat$ or group? or intervention? or investigat$ or method or participant? or procedure or study or trial?)).mp.

24. (control year? or experimental year? or control period? or experimental period?).mp.

25. ((strategy or strategies) adj3 (improv$ or education$)).mp.

26. ((single or doubl$ or tripl$ or treb$) adj3 (blind$ or mask$)).mp.

27. ("4 arm" or "four arm").mp.

28. exp case reports/ or "case report?".ti,ab.

29. or/14-28

30. 13 or 29

31. exp animals/ not humans.sh.

32. 30 not 31

33. 9 and 32

34. remove duplicates from 33

**PsycINFO**

| S36 | S6 AND S11 AND S35 |
| --- | --- |
| S35 | S33 NOT S34 |
| S34 | PO (animal NOT (human OR inpatient OR outpatient)) |
| S33 | S31 NOT S32 |
| S32 | SU "cluster analysis" |
| S31 | S15 OR S30 |
| S30 | S16 OR S17 OR S18 OR S19 OR S20 OR S21 OR S22 OR S23 OR S24 OR S25 OR S26 OR S27 OR S28 OR S29 |
| S29 | TI (case W1 (report OR reports)) OR AB (case W1 (report OR reports)) |
| S28 | "4 arm" OR "four arm" |
| S27 | (single OR doubl* OR tripl* OR treb*) W3 (blind* OR mask*) |
| S26 | AB ((strategy OR strategies) N3 (improv* OR education*)) |
| S25 | TI ((strategy OR strategies) N3 (improv* OR education*)) |
| S24 | AB ("control year" OR "experimental year" OR "control period" OR "experimental period") |
| S23 | TI ("control year" OR "experimental year" OR "control period" OR "experimental period") |
| S22 | AB (control N3 (area OR cohort OR compare* OR condition OR design OR group OR intervention OR participant OR study)) |
| S21 | AB ("time points" N3 (over OR multiple OR three OR four OR five OR six OR seven OR eight OR nine OR ten OR eleven OR twelve OR month OR hour OR day OR "more than")) |
| S20 | AB ((clinical OR epidemiologic OR evaluation OR validation) W2 (study OR studies OR trial)) |
| S19 | TI ((clinical OR epidemiologic OR evaluation OR validation) W2 (study OR studies OR trial)) |
| S18 | TI ( case* W3 (comparison* OR control* OR series) ) OR AB ( case* W3 (comparison* OR control* OR series) ) |
| S17 | AB (("before and after" OR case* OR cohort OR comparative OR controlled OR "cross section*" OR "follow up" OR longitudinal OR multicenter OR observation* OR prospective OR quasicontrol* OR quasi-control* OR quasiexperiment* or quasi-experiment* OR quasirandom* OR "record linkage" OR retrospective OR "time series") W3 (analy* OR design OR evaluat* OR investigat* OR method OR procedure OR study OR studies OR trial)) |
| S16 | TI (("before and after" OR case* OR cohort OR comparative OR controlled OR "cross section*" OR "follow up" OR longitudinal OR multicenter OR observation* OR prospective OR quasicontrol* OR quasi-control* OR quasiexperiment* or quasi-experiment* OR quasirandom* OR "record linkage" OR retrospective OR "time series") W3 (analy* OR design OR evaluat* OR investigat* OR method OR procedure OR study OR studies OR trial)) |
| S15 | S12 OR S13 OR S14 |
| S14 | TX (randomized controlled OR randomised controlled) |
| S13 | TX ( (randomiz* OR randomis* OR controlled OR placebo OR blind* OR unblind* OR "parallel group" OR crossover OR cross-over OR cluster OR "head to head") N3 (analy* OR design OR evaluat* OR investigat* OR method OR procedure OR study OR studies OR trial) ) |
| S12 | DE "Randomized Controlled Trials" OR DE "Randomized Clinical Trials" |
| S11 | S9 OR S10 |
| S10 | (female or girl or girls or women or woman) N3 (circumcis* or "genital cut*" or "genital mutilat*" |
| S9 | S7 AND S8 |
| S8 | (DE "Female Genitalia") OR (DE "Human Females") |
| S7 | DE "Circumcision" |
| S6 | S1 OR S2 OR S3 OR S4 OR S5 |
| S5 | DE "Health Literacy" OR DE "Health Knowledge" OR DE "Health Promotion" OR DE "Health Information" OR DE "Health Awareness" OR DE "Client Education" OR DE "Health Education" DE "Public Health Campaigns" OR DE "Sex Education" |
| S4 | DE "Medical Education" OR DE "Psychiatric Training" OR DE "Nursing Education" OR DE "Communication" |
| S3 | DE "Client Attitudes" OR DE "Client Satisfaction" OR DE "Community Attitudes" OR DE "Counselor Attitudes" OR DE "Cultural Attitudes" OR DE "Female Attitudes" OR DE "Health Personnel Attitudes" OR DE "Therapist Attitudes" OR DE "Male Attitudes" OR DE "Parental Attitudes" OR DE "Parental Expectations" OR DE "Psychotherapist Attitudes" OR DE "Sexual Attitudes" |
| S2 | inform* or educat* or counsel* or communicati* or promoti* or program or campaign or initiative or attitud* or knowledge* |
| S1 | DE "Counseling" OR DE "Community Counseling" OR DE "Cross Cultural Counseling" OR DE "Educational Counseling" OR DE "Group Counseling" OR DE "Marriage Counseling" OR DE "Conjoint Therapy" OR DE "Microcounseling" OR DE "Multicultural Counseling" OR DE "Pastoral Counseling" OR DE "Peer Counseling" OR DE "Premarital Counseling" OR DE "Psychotherapeutic Counseling" OR DE "Family Therapy" OR DE "Rehabilitation Counseling" OR DE "School Counseling" OR DE "Conjoint Therapy" OR DE "Strategic Family Therapy" OR DE "Structural Family Therapy" |

**SCOPUS**

(TITLE-ABS-KEY(inform* OR educat* OR counsel* OR communicati* OR promoti* OR program OR campaign OR initiative OR attitud* OR knowledge*)) AND (TITLE-ABS-KEY((female or girl or girls or women or woman) W/3 (circumcis* or "genital cut*" or "genital mutilat*"))) AND ((TITLE-ABS((randomiz* OR randomis* OR controlled OR placebo OR blind* OR unblind* OR "parallel group" OR crossover OR "cross over" OR cluster OR "head to head") W/4 (analy* OR design OR evaluat* OR investigat* OR method OR procedure OR study OR studies OR trial))) OR ((( TITLE-ABS(("before and after" OR case* OR cohort OR comparative OR "cross section*" OR "follow up" OR longitudinal OR multicenter OR observation* OR prospective OR quasicontrol* OR "quasi control*" OR quasiexperiment* or "quasi experiment*" OR quasirandom* OR "quasi random*" OR "record linkage" OR retrospective OR "time series") W/4 (analy* OR design OR evaluat* OR investigat* OR method OR procedure OR study OR studies OR trial))) OR (TITLE-ABS(case* W/3 (comparison* OR control* OR series))) OR (TITLE-ABS((clinical OR epidemiologic OR evaluation OR validation) PRE/3 (study OR studies OR trial))) OR (ABS("time points" W/3 (over OR multiple OR three OR four OR five OR six OR seven OR eight OR nine OR ten OR eleven OR twelve OR month OR hour OR day OR "more than"))) OR (ABS(control W/3 (area OR cohort OR compare* OR condition OR design OR group OR intervention OR participant OR study))) OR (TITLE-ABS("control year" OR "experimental year" OR "control period" OR "experimental period")) OR (TITLE-ABS((strategy OR strategies) W/2 (improv* OR education*)))) OR (TITLE-ABS-KEY((single OR doubl* OR tripl* OR treb*) PRE/3 (blind* OR mask*))) OR (TITLE-ABS-KEY("4 arm" OR "four arm")) OR (TITLE-ABS("case report*"))))

**Web of Science**

1: (female or girl or girls or women or woman) NEAR/3 (circumcis* or "genital cut*" or "genital mutilat*") (Topic)

2: TS=(inform* or educat* or counsel* or communicati* or promoti* or program or campaign or initiative or attitud* or knowledge*)

3: #1 AND #2

4: "case report" (Topic)

5: TI=(trial$) OR AB=("time points" NEAR/3 (over OR multiple OR three OR four OR five OR six OR seven OR eight OR nine OR ten OR eleven OR twelve OR month* OR hour$ OR day$ OR "more than"))

6: TS=(randomi?ed OR placebo OR randomly OR cohort*)

7: TS=(((clinical OR comparative OR controlled OR evaluation OR multicenter OR "multi-center" OR multicentre OR "multi-centre" OR validation) NEAR/3 (study OR studies OR trial$)))

8: TS=((case* NEAR/3 (comparison* OR control* OR series)))

9: TS=((("before and after" OR "before-and-after" OR case* OR "cross-section*" OR "cross section*" OR "follow up" OR "follow-up" OR longitudinal OR observation* OR prospective OR "record-linkage" OR "record linkage" OR retrospective OR "time-series" OR "time series") NEAR/3 (analy* OR design OR evaluat* OR investigat* OR method OR procedure OR study OR studies OR trial$)))

10: TS=(("quasi-experiment*" OR quasiexperiment* OR "quasi experiment*" OR "quasi random*" OR "quasi-random*" OR quasirandom* OR "quasi control*" OR "quasi-control*" OR quasicontrol*))

11: TS=((control* NEAR/4 (analy* OR area OR cohort$ OR compar* OR condition OR design OR evaluat* OR group$ OR intervention$ OR investigat* OR method OR participant$ OR procedure OR study OR trial$)))

12: TS=((control year$ OR experimental year$ OR control period$ OR experimental period$))

13: TS=(((strategy OR strategies) NEAR/3 (improv* OR education*)))

14: TS=(((single OR doubl* OR tripl* OR treb*) NEAR/3 (blind* OR mask*)) OR ("4 arm" OR "four arm"))

15: #4 OR #5 OR #6 OR #7 OR #8 OR #9 OR #10 OR #11 OR #12 OR #13 OR #14

16: #3 AND #15
