# Supplementary material for: Capacity building for healthcare workers on preventing and managing female genital mutilation: Impact on knowledge, attitudes, skills, and quality of care—A systematic review
Source: Int J Gynaecol Obstet. 2026 Jan 26;172(Suppl 1):48–64. doi: 10.1002/ijgo.70757 (PMC12833632; doi:10.1002/ijgo.70757)
Supplement: Supplementary file 2 — Table S2: a‐e: GRADE tables. [file IJGO-172-48-s001.docx]

**Table S2a: [Training of healthcare providers plus provision of IEC materials] compared to [provision of IEC materials only] for [improving health care provider's knowledge, attitude, skills, quality of care for girls and women living with FGM]**

| **Certainty assessment** | | | | | | | **№ of patients** | | **Effect** | | **Certainty** | |
| --- | --- | --- | --- | --- | --- | --- | --- | --- | --- | --- | --- | --- |
| **№ of studies** | **Study design** | **Risk of bias** | **Inconsistency** | **Indirectness** | **Imprecision** | **Other considerations** | **Provision of IEC materials and person-centered communication training package on FGM knowledge & prevention** | **Provision of IEC materials** | **Relative (95% CI)** | **Absolute (95% CI)** |  |  |
| **Knowledge of FGM** | | | | | | | | | | | |  |
| 1 | randomised trials | serious^a^ | not serious | not serious | not serious | none | The mean scores of FGM-related knowledge were higher among antenatal care (ANC) providers in the intervention arm (2.5, 95% Cl: 2.2-2.7) compared to the control arm (1.6, 95% Cl: 1.5-1.8) (P = <0.001)). | | | | ⨁⨁⨁◯ Moderate^a^ | |
| **Providing appropriate FGM-related prevention and care services** | | | | | | | | | | | |  |
| 1 | randomised trials | serious^a^ | not serious | not serious | not serious | none | One study reported that ANC providers in the intervention arm (6.2, 95% Cl 5.9-6.6) had higher scores than those in the control arm (3.7, 95% Cl 3.2-4.1) with a difference in the mean score of 2.5, 95% Cl:2.0 to 3.2; p<0.001. | | | | ⨁⨁⨁◯ Moderate^a^ | |
| **Confident in the knowledge to provide FGM prevention and care services** | | | | | | | | | | | |  |
| 1 | randomised trials | serious^a^ | not serious | not serious | not serious | none | 113/115 (98.3%) | 104/117 (88.9%) | **OR 7.06** (1.56 to 32.04) | **94 more per 1,000** (from 37 more to 107 more) | ⨁⨁⨁◯ Moderate^a^ | |
| **Appropriate interpersonal communication skills** | | | | | | | | | | | |  |
| 1 | randomised trials | serious^a^ | not serious | not serious | not serious | none | 82/115 (71.3%) | 68/117 (58.1%) | **OR 1.79** (1.04 to 3.09) | **132 more per 1,000** (from 10 more to 230 more) | ⨁⨁⨁◯ Moderate^a^ | |
| **Less supportive attitude towards FGM** | | | | | | | | | | | |  |
| 1 | randomised trials | serious^a^ | not serious | not serious | serious^b^ | none | 84/115 (73.0%) | 85/117 (72.6%) | **OR 1.02** (0.57 to 1.82) | **4 more per 1,000** (from 124 fewer to 103 more) | ⨁⨁◯◯ Low^a,b^ | |
| **Not supportive of FGM** | | | | | | | | | | | |  |
| 1 | randomised trials | serious^a^ | not serious | not serious | serious^b^ | none | 110/115 (95.7%) | 114/117 (97.4%) | **OR 0.58** (0.14 to 2.48) | **18 fewer per 1,000** (from 133 fewer to 15 more) | ⨁⨁◯◯ Low^a,b^ | |
| **Not supportive of medicalized FGM** | | | | | | | | | | | |  |
| 1 | randomised trials | serious^a^ | not serious | not serious | serious^b^ | none | 114/115 (99.1%) | 116/117 (99.1%) | **OR 0.98** (0.06 to 15.90) | **0 fewer per 1,000** (from 117 fewer to 8 more) | ⨁⨁◯◯ Low^a,b^ | |

**CI:** confidence interval; **OR:** odds ratio

#### Explanations

a. downgraded by one for risk of bias: Blinding of outcome assessment was unclear; Possibility of selection bias because clinical rotation schedule was used to determine enrollment

b. Confidence interval includes the null

References

Balde M, Ndavi P, Oyaro V, Soumah A, Esho Tammary, King’oo J et al. Cluster randomized trial of a health system strengthening approach applying person-centered communication for the prevention of female genital mutilation in Guinea, Kenya, and Somalia. BMJ Open. 2024;14(7):e078771 (https://doi.org/10.1136/bmjopen-2023-078771).

**Table S2b: [Educational intervention (Face-to-face; group discussion, hands-on demonstration)] compared to [No intervention] for healthcare provider's knowledge, attitude, skills, quality of care for girls and women living with FGM**

| **Certainty assessment** | | | | | | | **№ of patients** | | **Effect** | | **Certainty** |
| --- | --- | --- | --- | --- | --- | --- | --- | --- | --- | --- | --- |
| **№ of studies** | **Study design** | **Risk of bias** | **Inconsistency** | **Indirectness** | **Imprecision** | **Other considerations** | **Educational intervention (Face-to-face; group discussion, hands-on demonstration)** | **No intervention** | **Relative (95% CI)** | **Absolute (95% CI)** |  |
| **Knowledge of any type of FGM** | | | | | | | | | | | |
| 1^1^ | observational studies | serious^a,b^ | not serious | not serious | serious^d^ | none | 56/59 (94.9%) | 40/49 (81.6%) | **OR 4.20** (1.07 to 16.50) | **133 more per 1,000** (from 10 more to 170 more) | ⨁◯◯◯ Very low |
| **Disagreed that FGM poses no health risks if carried out in a hygienic environment** | | | | | | | | | | | |
| 1^1^ | observational studies | serious^a,b^ | not serious | not serious | serious^d^ | none | 31/58 (53.4%) | 32/48 (66.7%) | **OR 0.57** (0.26 to 1.27) | **134 fewer per 1,000** (from 134 fewer to 51 more) | ⨁◯◯◯ Very low |
| **Provider's willingness to lead anti-FGM campaigns** | | | | | | | | | | | |
| 1^1^ | observational studies | serious^a,b^ | not serious | not serious | serious^d^ | none | 54/59 (91.5%) | 42/49 (85.7%) | **OR 1.80** (0.53 to 6.08) | **58 more per 1,000** (from 96 fewer to 116 more) | ⨁◯◯◯ Very low |
| **Knowledge (ability to identify FGM-related complications** | | | | | | | | | | | |
| 1^1^ | observational studies | serious^a,b^ | not serious | not serious | serious^d^ | none | The knowledge of at least three immediate complications increased from 40% to 86%, and knowledge of subsequent complications from 49% to 72%. To a lesser degree, respondents from the control study sites also registered improvement in their knowledge of FGC complications. The total of 61% aware of possible consequences during the baseline study went to 73% in the follow-up study | | | | ⨁◯◯◯ Very low |
| **Improved communication skills and confidence to provide FGM prevention and care to their clients** | | | | | | | | | | | |
| 1^1^ | observational studies | serious^a^ | not serious | not serious | serious^c^ | none | One study reported that (96%) of the service providers who used the flip chart said that it enabled them to better communicate messages. Among the service providers questioned, 38% found that the flip chart was not constraining. Among the 63% who thought they were constraining, 56% still found it useful (Diop 1998).  The study also reported that among the total number of health agents who had received IEC training, more than half were unwilling to address FGC issues, compared to 1/3 of those who had not been trained in communication techniques. | | | | ⨁◯◯◯ Very low |

**CI:** confidence interval; **OR:** odds ratio

#### Explanations

a. Possible reporting bias

b. Possible selection bias

c. Small sample size

d. Small sample size (N=108); Wide confidence intervals that include the null

#### References

1.Diop, NJ.,Traore F,Diallo H,Traore O,Toure HA,Haidara A,et al.. Study of the effectiveness of training Malian social and health agents in female genital cutting issues and in educating their clients. Bamako: Division of Family and Community Health, Population Council, Association for the Support and Development of Population Activities, Republic of Mali; 2007.

**Table S2c: [A virtual, dramatization simulation session with a standardized patient with FGM] compared to [No intervention] for [improving health care provider's knowledge, attitude, skills, quality of care for girls and women living with FGM]**

| **Certainty assessment** | | | | | | | **№ of patients** | | **Effect** | | **Certainty** |
| --- | --- | --- | --- | --- | --- | --- | --- | --- | --- | --- | --- |
| **№ of studies** | **Study design** | **Risk of bias** | **Inconsistency** | **Indirectness** | **Imprecision** | **Other considerations** | **A virtual, dramatization simulation session with a standardized patient with FGM** | **No intervention** | **Relative (95% CI)** | **Absolute (95% CI)** |  |
| **Knowledge of FGC** | | | | | | | | | | | |
| 1^1^ | observational studies | serious^a^ | not serious | not serious | serious^b^ | none | There was an improvement in knowledge of FGC with a mean score of 9.50 (SD=1.345) in the intervention group compared to 6.76 (SD=4.640) in the control group; with a mean difference of 2.74 (0.59 – 4.89). | | | | ⨁◯◯◯ Very low |
| **Knowledge that the consequences of FGM on health are numerous** | | | | | | | | | | | |
| 1^1^ | observational studies | serious^a^ | serious | not serious | serious^b,c^ | none | 11/14 (78.6%) | 18/20 (90.0%) | **OR 0.41** (0.06 to 2.84) | **113 fewer per 1,000** (from 549 fewer to 62 more) | ⨁◯◯◯ Very low |
| **Attitude about their support for FGM, FGM medicalization & reinfibulation** | | | | | | | | | | | |
| 1^1^ | observational studies | serious^a^ | serious | not serious | serious^b^ | none | The authors reported a 26.5% increase in the number of participants that thought that FGC should not be medicalized on the posttest. | | | | ⨁◯◯◯ Very low |

**CI:** confidence interval; **OR:** odds ratio

#### Explanations

a. Possible reporting bias

b. Small sample size (n=35)

c. 95% CI intervals includes the null

#### References

1.Hess, R. F.,Ross,R.,Wyss,L.,& Donnenwirth,J. A.. Nursing students' knowledge gained about female genital cutting/mutilation through dramatization simulation with a standardized patient: A quasi-experimental study. Nurse Education Today; 2022.

**Table S2d: Educational intervention (Face-to-face; group discussion, hands-on demonstration) (No comparison arm) for healthcare provider's knowledge, attitude, skills, quality of care for girls and women living with FGM**

| **Certainty assessment** | | | | | | | **Impact** | **Certainty** |
| --- | --- | --- | --- | --- | --- | --- | --- | --- |
| **№ of studies** | **Study design** | **Risk of bias** | **Inconsistency** | **Indirectness** | **Imprecision** | **Other considerations** |  |  |
| **Knowledge of FGC** | | | | | | | | |
| 3^1,2,3^ | observational studies | serious^a,b,c^ | not serious | not serious | serious^d^ | none | • One study (n = 11) reported an increase from a mean of 2.36 to 4.18 after the intervention (Jacoby & Smith 2013).  • Another study (n = 49) reported an increase in the number of participants who knew the number of the types of FGM from 17 (35%) to 41 (85%), X2 (6, N = 97) = 29.10, p <.001 (Elliot 2016).  • Another study (n =26) reported an increase in the knowledge of FGM/C-related contents (64% at baseline versus 96.2% post-training (t(25 = 7.408, p < 0.001); knowledge on the types of FGM/C (84.6% at baseline versus 100% post-training (t(25) = 2.132, p=0.043); and knowledge that FGM/C is a global health problem and threat to maternal-child-infant health (96.2% versus 100%) (Kimani 2018). | ⨁◯◯◯ Very low |
| **Confidence and ability to identify FGM-related complications** | | | | | | | | |
| 1^1^ | observational studies | serious^a,b,e^ | not serious | not serious | serious^d^ | none | One study (n=26) reported an improvement in participants’ knowledge scores on FGM/C-related health consequences (immediate physical (p = 0.003), gynecological (p = 0.001), obstetric (p = 0.022), sexual (p < 0.001), and social harms (p < 0.001). There was no statistically significant difference in knowledge scores regarding FGM/C-related psychological complications post-intervention (Kimani 2018) | ⨁◯◯◯ Very low |
| **Attitude about their support for FGM, FGM medicalization & reinfibulation:** | | | | | | | | |
| 2^1,3^ | observational studies | serious^a,b,e,f^ | serious | not serious | serious^d^ | none | • One study (n=49) reported no significant difference in response towards allowing mild circumcision on girls under 18, circumcision on consenting adult women, and reinfibulation after childbirth among participants, before and after the intervention (Elliot 2016).  • Another study (n=26) reported an improvement following training on questions like pledging never to perform FGM/C because it is not medically necessary and causes harm (t(25) = -5.000, p < 0.001) (Kimani 2018).  • All the participants agreed that FGM/C violates women's and girls' human rights (p = 0.043), and FGM performed by medical personnel is not safer (Kimani 2018). The study reported significant improvement on the health care provider to resist infibulation when requested by a woman supposedly under pressure from her husband (P<0.001).  • Participants’ knowledge was reported to be poor to moderate on the importance of the need to leave the woman deinfibulation (Kimani 20018) | ⨁◯◯◯ Very low |
| **Communication skills and confidence to provide FGM prevention and care to their clients** | | | | | | | | |
| 1^2^ | observational studies | not serious | not serious | not serious | serious^g^ | none | • One study reported an increase in the mean confidence level (counseling women with type III FGM/C) of participants from 2.36 to 4.09. The mean confidence level of healthcare providers increased from 1.54 to 3.54 following the practice of deinfibulation and repair using simulated pelvic models (Jacoby & Smith 2013) | ⨁◯◯◯ Very low |

#### Explanations

a. Bias due to confounding likely (Kimani 2018_Difference in the educational qualifications of participants)

b. Bias in the measurement of outcome (Kimani 2018)

c. Possible reporting bias (Jacoby & Smith 2013, Elliot 2016, Kimani 2018)

d. Small sample size

e. Possible reporting bias (Kimani 201)

f. Possible reporting bias (Elliot 2016)

g. Possible reporting bias (Jacoby & Smith 2013)

#### References

1.Kimani S, Esho T,Kimani V,Muniu S,Kamau J,Kigondu C et al.. Female genital mutilation/cutting: Innovative training approach for nurse-midwives in high prevalent settings. Obstetrics & Gynecology Int.; 2018.

2.A, Jacoby,SD,&,Smith. Increasing Certified nurse-midwives' confidence in managing the obstetric care of women with female genital mutilation/cutting. J. Midwifery Womens Health; 2013.

3.Elliott C, Creighton SM,Barker M,Liao L. A brief interactive training for health care professionals working with people affected by "female genital mutilation": initial pilot evaluation with psychosexual therapist. Sexual and Relationship Therapy; 2016.

**Table S2e: [Virtual training] for [improving health care provider's knowledge, attitude, skills, quality of care for girls and women living with FGM]**

| **Certainty assessment** | | | | | | | **Impact** | **Certainty** |
| --- | --- | --- | --- | --- | --- | --- | --- | --- |
| **№ of studies** | **Study design** | **Risk of bias** | **Inconsistency** | **Indirectness** | **Imprecision** | **Other considerations** |  |  |
| **Knowledge of FGM** | | | | | | | | |
| 1^1^ | observational studies | serious^a^ | not serious | not serious | serious^b^ | none | The mean (SD) FGC knowledge scores increased from 32.95 (2.977) at baseline to 38.49 (2.8953) post-intervention (MD: 5.54 (SD:4.5; (-6.495 - -4.583) p <0.001 (Barnawi 2018) | ⨁◯◯◯ Very low |
| **Management of patients with FGM-related complications** | | | | | | | | |
| 1^2^ | observational studies | serious^c^ | not serious | not serious | serious^d^ | none | The mean number of FGC complications that were treated on site increased by 26% (Newman 2003) | ⨁◯◯◯ Very low |
| **Communication skills and confidence to provide FGM prevention and care to their clients** | | | | | | | | |
| 2^1,2^ | observational studies | serious^a,c^ | serious | not serious | serious^b,d^ | none | • There was an increase in the level of readiness of students to provide competent care to manage FGC from (M (SD) = 15.7629 (1.88) pretest scores to 18.567 (1.89) at the posttest. (mean difference:- 2.80 (-3.37 - -2.24) p <0.001) (Barnawi 2018).  • After the intervention, providers were three times more likely to ask pregnant women if they have FGC complications that might affect birthing (Newman 2003). | ⨁◯◯◯ Very low |
| **Attitude about their support for FGM, FGM medicalization & reinfibulation** | | | | | | | | |
| 1^1^ | observational studies | serious^a^ | not serious | not serious | serious^b^ | none | The mean (SD) FGC attitude scores increased from 49.42 (5.475) at baseline to 54.89 (5.902) post-intervention (MD: 5.47 (SD:8.3; (-7.18 - -3.77) p <0.001 (Barnawi 2018) | ⨁◯◯◯ Very low |
| **Skills on providing FGM-related education and counseling for communities in outreach services** | | | | | | | | |
| 1^2^ | observational studies | serious^c^ | not serious | not serious | serious^d^ | none | • Before the intervention, counseling on FGC was non-existent, but following the intervention, 414 female prenatal care clients received counseling about abandoning the practice, with nearly three-quarters of providers passing the counseling skills performance test.  • Providers were also present at 714/1187 community outreach sessions and 473 educational sessions in the health centers on the negative effects of FGC | ⨁◯◯◯ Very low |

#### Explanations

a. Possible biases due to departure from intended interventions, missing data, and reporting result (Barnawi 2018)

b. Small sample size (n=86)

c. Possible biases due to missing data and reporting result (Newman 2003)

d. Small sample size (n=120)

#### References

1.N, Barnawi. The effects of a digital educational intervention on undergraduate nursing students' attitudes, knowledge and self-efficacy with female genital cutting. Graduate Dissertations and Theses ; 2018.

2.Newman C, Nelson D.. Couseling and Advocacy to Abandon Female Genital Cutting. PRIME; 2003.
